# Lonicerae flos and turmeric extracts alleviate necrotic enteritis in broilers by modulating gut-liver health and microbiota

**DOI:** 10.1186/s40104-025-01246-1

**Published:** 2025-08-08

**Authors:** Xingbo Liu, Yunru Ji, Huiyuan Lv, Zhong Wang, Zengpeng Lv, Yuming Guo, Wei Nie

**Affiliations:** 1 State Key Laboratory of Animal Nutrition and Feeding, College of Animal Science and Technology, China Agricultural University, Beijing, 100193 China; 2 Beijing Centre Biology Co., Ltd., Beijing, 102600 China

**Keywords:** Chlorogenic acid, Curcumin, Microbiota, Mitochondrial dysfunction, Necrotic enteritis, Secondary liver injury

## Abstract

**Background:**

Necrotic enteritis (NE) can cause intestinal barrier dysfunction in broilers, leading to secondary liver injury (SLI). In this process, the gut-liver axis plays a crucial role. Lonicerae flos and turmeric extracts (LTE), containing chlorogenic acid and curcumin, have been reported to possess anti-inflammatory, and antioxidant properties. Based on these potential biological benefits, this study aims to investigate the reparative effects of LTE on the intestinal barrier dysfunction in NE-infected broilers and assess its therapeutic efficacy in alleviating SLI. By elucidating the regulatory mechanisms of LTE on gut-liver axis health, this research provides new insights into the prevention and treatment of NE in broilers.

**Results:**

LTE improved body weight and average daily gain while reducing intestinal lesion scores, coccidia oocysts, and *Clostridium perfringens* counts in NE broilers (*P* < 0.05). LTE enhanced intestinal morphology and up-regulated the expression of tight junction protein genes (*CLDN1*, *TJP1*) and *MUC2*, suppressed pro-inflammatory cytokine and myeloperoxidase (MPO) levels, and minimized endotoxin (ET) accumulation in NE broilers (*P* < 0.05). Furthermore, LTE alleviated oxidative stress in ileal cells and protected mitochondrial structure and function in NE broilers. NE infection induced intestinal permeability in broilers, leading to increased serum pro-inflammatory cytokines and intestinal-derived endotoxin levels, which caused liver damage. LTE significantly reduced liver pathologic damage, pro-inflammatory cytokine levels, aspartate transaminase, alanine aminotransferase, and ROS levels in NE broilers (*P* < 0.05). Additionally, 16S rRNA sequencing revealed that NE significantly increased the relative abundance of *Barnesiella* and decreased the relative abundance of *Bacteroidota*, *Desulfobacterota* and *Bacteroides* in the cecum of broilers. LTE enhanced intestinal microbiota diversity and reduced the segregation of intestinal microbiota induced by NE infection.

**Conclusions:**

In summary, LTE can alleviate NE and SLI by modulating the microbiota, inhibiting inflammation and oxidative stress, and ameliorating mitochondrial dysfunction, thereby enhancing gut-liver axis health and growth performance.

## Background

Necrotic enteritis (NE) is a common intestinal disease in the poultry industry, primarily caused by *Clostridium perfringens* [1]. The subclinical form of NE is prevalent, characterized by inflammation of the intestinal mucosa, with affected animals showing signs of lethargy and reduced appetite. The insidious nature of this disease results in significant economic losses to the poultry industry [2]. Factors such as coccidial infection, nutritional imbalances, mycotoxins, physiological stress, and other diseases can alter the gut barrier function and immune status of broilers, increasing the likelihood of NE occurrence [3]. Growing evidence suggests that the pathogenesis of NE is related to intestinal barrier dysfunction [4, 5]. Oxidative stress caused by reactive oxygen species (ROS) exacerbates the development of NE [6, 7]. The disruption of the intestinal barrier allows toxic and harmful substances from the gut lumen to invade, with gut-derived endotoxins being a key factor in inducing liver injury [8]. Liver injury can lead to disturbances in bile acid circulation and the release of pro-inflammatory cytokines, further exacerbating the development of NE [9]. Therefore, regulating the gut-liver axis is crucial for the treatment and prevention of NE.

The impact of gut microbiota on host intestinal health is multifaceted, including nutrient metabolism [10], immune regulation [11], and energy metabolism [12]. Several studies have shown that dysbiosis of the gut microbiota is closely associated with NE broilers [13, 14]. It has been reported that NE significantly increases the abundance of bacteria associated with inflammation, obesity, and depression, while significantly reducing the abundance of short-chain fatty acid (SCFA)-producing bacteria in the cecum [13]. Moreover, the interaction between the gut microbiota and host oxidative stress and mitochondria of intestinal epithelial cells is crucial for maintaining intestinal homeostasis [15]. Metabolites produced by gut microbiota, such as SCFAs, hydrogen sulfide, and lipopolysaccharides, can influence ROS levels and mitochondrial metabolism [16]. Mitochondrial dysfunction in intestinal epithelial cells can trigger metabolic imbalances, leading to changes in the oxygen environment within the gut lumen and alterations in the gut microbiota structure [17]. Therefore, seeking safe and effective feed additives that can improve gut barrier function, modulate the gut microbiota, and alleviate oxidative stress has become a feasible strategy for the treatment and prevention of NE in broilers.

Lonicerae flos and turmeric extracts (LTE) are a natural complex plant extract with chlorogenic acid (CA) and curcumin (CUR) as the bioactive components. Previous studies have demonstrated its anti-inflammatory, antioxidant, and immunomodulatory properties [18]. Stress and inflammation are significant pathological features of NE, this prompted us to investigate whether LTE can ameliorate NE. CA has been shown to possess hepatoprotective effects [19], modulate the gut microbiota [20], and address mitochondrial dysfunction [21]. Similarly, CUR's interaction with the gut microbiota positions it as a potential therapeutic agent for intestinal barrier dysfunction and related diseases [22]. Additionally, CUR has been demonstrated to reduce oxidative stress and alleviate diseases associated with mitochondrial dysfunction [23]. These findings suggest that CA and CUR can regulate both mitochondria and the gut microbiota, indicating that LTE, which contains these compounds, may also possess these beneficial functions.

Based on the aforementioned findings, we hypothesized that LTE may alleviate NE by improving gut-liver health and modulating the gut microbiota, and reducing inflammation and oxidative stress. To address this, we utilized 16S rRNA sequencing to identify the gut microbiota and analyzed the effects of LTE on growth performance, intestinal barrier structure and function, mitochondrial structure and function, antioxidant capacity, and liver damage in NE broilers. The results of this study will provide new insights into the prevention and treatment of NE in broilers.

## Materials and methods

### Animals, diets and experimental design

A total of 324 1-day-old male Arbor Acres (AA) broilers were selected for the experiment and randomly divided into 3 treatment groups with 6 replicates of 18 chickens each. The total trial period was 28 d, divided into 1–21 d and 22–28 d. The treatment groups were as follows: (i) NC group, unchallenged and without additive; (ii) PC group, challenged and without additives; and (iii) LTE300 group, challenged and dietary supplementation with 300 mg/kg LTE. LTE used in this experiment has been introduced by Ji et al. [18]. In brief, the LTE was provided by Beijing Centre Biology Co., Ltd. (Beijing, China), and the main active ingredients are CA and CUR. The contents of CA and CUR in the LTE are 100 mg/kg and 200 mg/kg, respectively, as provided by the manufacturer. Each kg of the basal diet in the LTE300 group contains 300 mg of LTE, providing 30 mg of chlorogenic acid and 60 mg of curcumin per kg diet. LTE were first mixed with the premix and then mixed with other ingredients by the principle of step-by-step premixing. The basal diets were formulated according to the Chinese Standard for Chicken Feeding (NY/T 33–2004) [24], and the basal diet composition and nutrient levels were shown in Table 1. The diets were analyzed for dry matter (DM, method 934.01) and crude protein (CP, method 990.03) following the AOAC procedures [25]. Calcium (GB/T 6436–2018) [26], and phosphorus (GB/T 6437–2018) [27] levels were determined according to the Chinese national standards. The gross energy (GE) of diets was determined using an adiabatic bomb calorimeter (C 2000; IKA, Guangzhou, China) with benzoic acid as the standard. Broilers were fed and watered ad libitum. Room temperature and lighting can be adjusted, and the temperature was 33 °C for the first 3 d, then decreased to 23 °C at a rate of 1 °C every 2 d. The average relative humidity was maintained at approximately 70% in the first 3 d and thereafter maintained between 55% and 65%. Broilers were kept under 23 h of light and 1 h of darkness in the first week, followed by 20 h of light and 4 h of darkness for the subsequent period.

**Table 1:** Composition and nutrient levels of the basal diet (as-fed basis)

Ingredient, %	Days 1–21	Days 22–28
Corn	59.09	61.78
Soybean meal (43%)	30.00	26.00
Cottonseed meal (46%)	3.00	3.00
Corn gluten meal (60%)	2.50	2.50
Dicalcium phosphate	1.55	1.35
Soybean oil	1.50	3.00
Limestone	1.35	1.35
0.5% Premix^1^	0.55	0.55
L-Lysine sulfate (70%)	0.29	0.30
*DL*-methionine	0.12	0.12
Choline chloride (60%)	0.05	0.05
Total	100.00	100.00
Analyzed nutrients		
Gross energy, MJ/kg	16.46	17.21
Dry matter, %	88.94	88.71
Crude protein, %	21.88	20.65
Calcium, %	0.78	0.67
Total phosphorus, %	0.62	0.57
Calculated nutrients		
Metabolizable energy, MJ/kg	12.13	12.66
Available phosphorus, %	0.40	0.35
Lysine, %	1.14	1.06
Methionine, %	0.44	0.42

^1^Premix supplied per kilogram of the diet: vitamin A 12,000 IU, vitamin D_3_ 2,700 IU, vitamin E 50 mg, vitamin K_3_ 3.0 mg, vitamin B_1_ 3.0 mg, vitamin B_2_ 10.50 mg, vitamin B_6_ 6.20 mg, vitamin B_12_ 0.03 mg, biotin 0.22 mg, folic acid 1.50 mg, pantothenic acid 18 mg, niacinamide 60 mg, iron 80 mg, copper 8 mg, zinc 60 mg, manganese 60 mg, iodine 0.35 mg, selenium 0.15 mg

### Necrotic enteritis challenge

Necrotic enteritis was induced in broilers following the method described by Wu et al. [28]. On d 12, broilers in the PC and LTE300 groups were orally administered 1 mL of a 20-fold concentrated coccidia vaccine, while those in the NC group received 1 mL of sterile PBS solution orally. From 17 to 20 d, broilers in the PC and LTE300 groups were continuously given 1 mL of freshly prepared *Clostridium perfringens* (CVCC52) suspension at a concentration of 5 × 10^8^ CFU/mL for 4 d. In contrast, broilers in the NC group were administered sterile *Clostridium perfringens* liquid medium (Qingdao Haibo Biotechnology Co., Ltd., China). The preparation method for the *Clostridium perfringens* suspension is as follows: First, the *Clostridium perfringens* strain, stored at −20 °C, is quickly thawed and inoculated onto a tryptone-sulfite-cycloserine agar plates (Qingdao Hope Bio-Technology Co., Ltd., Qingdao, China). It is then incubated under anaerobic conditions at 37 °C for approximately 18 h to activate the strain. Subsequently, the activated *Clostridium perfringens* strain is inoculated into 300 mL of sterile reinforced clostridial medium (Qingdao Haibo Biotechnology Co., Ltd., China) and further incubated anaerobically at 37 °C for approximately 18 h to obtain the *Clostridium perfringens* suspension.

### Growth performance

Body weight (BW) and feed weight of broilers were recorded on d 1, 21, and 28. The average daily gain (ADG), average daily feed intake (ADFI), feed conversion ratio (FCR), and mortality were calculated for the periods from 1 to 21 d, and 1 to 28 d. The number of dead broilers was recorded to calculate mortality and correct feed intake.

### Sample collection

On d 21 and 28, one broiler of near average weight was randomly selected per replicate. Blood was collected from the wing vein of broilers into a vacuum blood collection tube, centrifuged at 3,000 r/min for 15 min, and the serum was collected and stored at −20 °C. Subsequently, the broilers were bled to death from the jugular vein, the abdominal cavity was opened, and lesion scores for the duodenum, jejunum, ileum, and cecum were recorded. The liver was isolated weighed, and the liver index was calculated. A small sample of ileum tissue, approximately 1 mm^3^ in volume, was quickly collected and fixed in 2.5% glutaraldehyde for mitochondrial transmission electron microscopy analysis. Approximately 1 cm sections of mid-ileum and liver were taken and fixed in 4% paraformaldehyde solution for morphological and pathological observations and analysis. Liver and ileum tissues were collected in sterile centrifuge tubes and stored at −80 °C. Mitochondria were extracted from approximately 120 mg of fresh ileum tissue (collected within 1 h after euthanizing the broilers) from 28-day-old broilers, strictly following the instructions provided with the mitochondrial extraction kit (C3606, Beyotime Biotechnology Co., Ltd., Shanghai, China). The extracted mitochondrial samples were stored at −80 °C. Cecum contents were collected and stored at −80 °C for microbiota analysis.

### Intestinal lesion scoring

The duodenum, jejunum, ileum and cecum were scored according to the Dahiya et al. [29]. Lesion scoring scale (0–4) was as follows: 0 = normal intestinal appearance, no lesions; 0.5 = severely congested serosa and mesentery engorged with blood; 1 = thin walled and friable intestines with small red petechiae (> 5); 2 = focal necrotic lesions; 3 = patches of necrosis (1 to 2 cm long); and 4 = diffused necrosis typical of field cases.

### Coccidia oocysts count in feces

On d 17, 18, and 19, 200 g feces were collected from each replicate for subsequent coccidia oocyst counts after thorough mixing. The counting method followed Xu et al. [14]. Briefly, 2 g feces were placed in a conical vial, mixed with 60 mL of saturated saline solution, and the supernatant was transferred to a McMaster counting plate (Shanghai Institute of Parasitic Diseases, Chinese Academy of Agricultural Sciences, Shanghai, China). After allowing the mixture to stand for 5 min, oocysts were counted using an electron microscope (Leica DM750, Shanghai, China). The oocyst content per gram of feces (OPG) was calculated according to the following formula: OPG = total number of oocysts in the counting chamber × 200 × dilution.

### *Clostridium perfringens* count in the cecum

The bacterial culture method of Wu et al. [28] was referred to determine the bacterial counts in the cecum chyme at d 21 and 28. Briefly, 0.2 g cecum chyme was taken in a 5-mL sterile centrifuge tube, and the sample weight was recorded accurately. Nine times the volume of pre-cooled sterilised saline was added, and the mixture was vortexed thoroughly. Serial dilutions were made to obtain 10^–1^ to 10^–5^ dilutions. Next, 100 μL of each dilution was inoculated onto tryptone-sulfite-cycloserine agar plates (Qingdao Hope Bio-Technology Co., Ltd., Qingdao, China) and incubated anaerobically for 24 h at 37 °C. The colony-forming units (CFU) per gram of chyme were counted, and the results were expressed as lgCFU/g after log10 transformation.

### Liver index and pathological analysis

Liver index was calculated: liver index (%) = [liver weight (g)/live body weight (g)] × 100%. Liver samples fixed in 4% paraformaldehyde solution were subsequently embedded in paraffin, cut into thin slices and stained with hematoxylin and eosin (H&E), followed by observation of the morphological features of the liver tissues under an electron microscope (Leica DM750, Shanghai, China).

### Intestinal morphology and goblet cell count

Ileal samples fixed in 4% paraformaldehyde solution were subsequently embedded in paraffin, cut into thin sections and stained with H&E and Alcian blue-Periodic acid Schiff (AB-PAS). Ileal villus height (VH), villus width (VW), crypt depth (CD) and muscular height (MH) were determined under an electron microscope (Leica DM750, Shanghai, China), and the ratio of villus height to crypt depth (VH/CD) was calculated, as well as the number of goblet cells per 100 μm of villi.

### Analysis of AST and ALT levels

Aspartate aminotransferase (AST) and alanine aminotransferase (ALT) levels in serum of 28-day-old broilers were determined using an AST kit (C010-2-1, Nanjing Jiancheng Bioengineering Institute, Nanjing, China) and an ALT kit (C009-2-1, Nanjing Jiancheng Bioengineering Institute, Nanjing, China). Results were showed in U/L.

### Levels of endotoxin and inflammatory factor

Chicken enzyme-linked immunosorbent assay (ELISA) kits were used to determine the levels of interleukin-1β (IL-1β, MM-36910O1), interleukin-6 (IL-6, MM-0521O1) and tumour necrosis factor-α (TNF-α, MM-0938O1). In addition, endotoxin (ET) levels in serum, liver and cecum chyme were detected using an ELISA kit (MM-37108O1). All assay procedures were performed strictly according to the manufacturer’s instructions (Jiangsu Meimian Industrial Co., Ltd., Nanjing, China).

### Determination of antioxidant enzyme activities in ileum and mitochondria

Both ileal tissue and mitochondria were prepared as homogenised tissue at a weight:saline volume ratio of 1:9 and the supernatant was retained for subsequent analysis. Catalase (CAT, BC4780, Beijing Solarbio Science & Technology Co., Ltd., Beijing, China), total superoxide dismutase (T-SOD, A001-1-2, Nanjing Jiancheng Bioengineering Institute, Nanjing, China) and glutathione peroxidase (GSH-Px, A005-1-2, Nanjing Jiancheng Bioengineering Institute, Nanjing, China) levels were measured in the ileal tissues and mitochondria of broilers on d 28 by using commercially available kits. Protein concentrations in the supernatants were determined using the BCA protein concentration kit (P0012S, Beyotime Biotechnology Co., Ltd., Shanghai, China). The assays were performed in strict accordance with the kit instructions.

### Analysis of ROS, MPO and ATP levels

10% ileal, liver and mitochondrial tissue homogenates were prepared at a weight:saline volume ratio of 1:9. Reactive oxygen species (ROS, MM-60120O1, Jiangsu Meimian Industrial Co., Ltd., Nanjing, China) levels in ileum, liver and mitochondrial tissues were determined using an ELISA kit. In addition, myeloperoxidase (MPO) levels in ileal tissues were determined using a kit (A044-1-1, Nanjing Jiancheng Bioengineering Institute, Nanjing, China). Adenosine triphosphate (ATP) kit (A095-1-1, Nanjing Jiancheng Bioengineering Institute, Nanjing, China) was used to determine ATP levels in broiler mitochondrial tissues. The assay procedures were all carried out in strict accordance with the kit instructions.

### Mitochondrial transmission electron microscopy

Ileal samples fixed in 2.5% glutaraldehyde solution were prepared according to standard procedures for sample processing (Wuhan Servicebio Technology Co., Ltd., Wuhan, China). Then, the ultrastructure of mitochondria was observed and electron micrographs were captured under a transmission electron microscope (Hitachi TEM system), and images were randomly acquired at 5.0 k, 15.0 k, and 40.0 k magnification.

### Quantitative real-time PCR (qRT-PCR) analysis

Total RNA was extracted from the ileum and liver of 28-day-old broilers using RNAiso Plus reagent (Takara, Tokyo, Japan). The purity and concentration of total RNA were determined using a NanoDrop 2000 spectrophotometer (Thermo Fisher Scientific Inc., Walldorf, Germany). The cDNA was synthesised using PrimeScript^™^ RT reagent kit with gDNA Eraser (Takara, Tokyo, Japan). Finally, qRT-PCR was performed using TB Green^®^ Premix Ex Taq^™^ kit (Takara, Tokyo, Japan) and Applied Biosystems 7500 Fast Real-Time PCR System (Foster City, CA, USA). *β-actin* was used as an internal reference gene, and the relative expression of mRNA of the target gene was calculated using the 2^−ΔΔCt^ method. The primer information is shown in Table 2.

**Table 2:** Sequences of the oligonucleotide primers used for qRT-PCR

Gene names	Primers sequence (5′→3′)	Accession No.
*CLDN1*	F: AAGTGCATGGAGGATGACCA R: GCCACTCTGTTGCCATACCA	NM_001013611.2
*OCLN*	F: AGTTCGACACCGACCTGAAG R: TCCTGGTATTGAGGGCTGTC	NM_205128.1
*TJP1*	F: ACAGCTCATCACAGCCTCCT R: TGAAGGGCTTACAGGAATGG	XM_015278981.1
*MUC2*	F: TGGATACTTGCTCACACTTGG R: GTTAGCAAAGGCAGATCCTGT	XM_040701656.2
*Nrf2*	F: TTCGCAGAGCACAGATACTTC R: TGGGTGGCTGAGTTTGATTAG	NM_205117.1
*HO-1*	F: ACAACGCTGAAAGCATGTCCC R:AGATGAAGTACAGGGACGCC	NM_205344.1
*NQO1*	F: CGCACCCTGAGAAAACCTCT R: ACTGCAGTGGGAACTGGAAG	NM_001277619.1
*β-actin*	F: GAGAAATTGTGCGTGACATCA R: CCTGAACCTCTCATTGCCA	NM_205518.1

*F* Forward, *R* Reverse, *CLDN1* Claudin 1, *OCLN* Occludin, *TJP1* Tight junction protein 1, *MUC2* Mucin 2, *Nrf2* Nuclear erythroid-derived 2-related factor 2, *HO-1* Heme oxygenase-1, *NQO1* NAD(P)H quinone oxidoreductase-1

### 16S rRNA sequencing for microbiota analysis

The genomic DNA was extracted from approximately 0.3 g of cecal contents of 28-day-old broilers using the CTAB kit (Nobleryder, Beijing, China). PCR amplification of the V3–V4 variable region of the 16S rRNA gene was amplified by 341 F (5'-CCTAYGGGRBGCASCAG-3') and 806R (5'-GGACTACNNGGGTATCTAAT-3'). The PCR reaction conditions were: pre-denaturation at 98 °C for 1 min, 30 cycles (denaturation at 98 °C for 10 s, annealing at 50 °C for 30 s, and extension at 72 °C for 30 s), followed by stable extension at 72 °C for 5 min. The PCR products were detected by electrophoresis using a 2% agarose gel, and the qualified PCR products were purified by magnetic beads, quantified by enzyme labelling. TruSeq^®^ DNA PCR-Free Sample Preparation Kit was used for library construction. The constructed library was quantified by Qubit and Q-PCR, and after the library was qualified, NovaSeq6000 was used for on-line sequencing.

Raw FASTQ files were de-multiplexed using an in-house Perl script, and then quality-filtered by fastp (v0.22.0, https://github.com/OpenGene/fastp) and merged by FLASH (v1.2.11, http://ccb.jhu.edu/software/FLASH/) with the following criteria: (i) automatically detect and remove adapter sequences; remove reads with 15 or more N bases; (ii) remove reads with more than 50% low-quality bases (quality score ≤ 20); (iii) delete reads with an average quality score below 20 in a 4-base window; (iv) remove polyG tails; and (v) remove reads shorter than 150 bp. Then the optimized sequences were clustered into operational taxonomic units (OTUs) using UPARSE v7 algorithm (http://drive5.com/uparse/) with 97% sequence similarity level. The most abundant sequence for each OTU was selected as a representative sequence. Species annotation of OTU sequences was performed and analysed for species annotation using the Mothur method with the SSUrRNA database of SILVA138.1 (http://www.arb-silva.de/) to obtain taxonomic information and to count the community composition of each sample at each taxonomic level separately. Use MAFFT (v7.520, https://mafft.cbrc.jp/alignment/software/) software for fast multiple sequence alignment to obtain the phylogenetic relationships of all OTU representative sequences. Finally, normalize the data of each sample, using the sample with the smallest data amount as the reference for normalization. Subsequent Alpha diversity and Beta diversity analyses are based on the normalized data.

Data were analysed and visualised on the Metware cloud platform (https://cloud.metware.cn/). Phyloseq (v1.40.0) and vegan (v2.6.2) packages of R software (v4.2.0) were used to calculate Chao, Shannon, Simpson, and ACE indices, and to plot rarefaction curve and rank abundance curve. Similarity in microbial community structure between samples was tested using principal co-ordinates analysis (PCoA) and non-metric multidimensional scaling (NMDS) analysis based on the Bray–Curtis distance algorithm and combined with the ANOSIM non-parametric test to analyse whether differences in microbial community structure between groups were significant. ANOVA analysis was used to analyze the differences at the phylum and genus levels of the microbiota.

### Statistical analysis

Data were analyzed for the homogeneity of variances and normality using Levene’s and Shapiro–Wilk’s tests, respectively. Data that were heterogeneous or not normally distributed were analyzed using non-parametric Kruskal–Wallis test, and pairwise differences in rank sums were evaluated using selected comparisons tests. The normal data were tested for statistical significance using one-way ANOVA and Duncan’s post hoc test for pairwise comparisons. Data were analyzed by using SPSS statistical software (ver. 25.0 for Windows, SPSS Inc., Chicago, USA). The results expressed as mean ± standard error of the mean (SEM). Correlation analysis was conducted using Spearman's rank correlation coefficient. *P* < 0.05 indicating a significant difference and 0.05 ≤ *P* < 0.1 indicating a trend of difference.

## Results

### LTE improves growth performance and clinical manifestation in NE broilers

As shown in Table 3, compared with NC group, the BW on d 21 and 28, as well as the ADG from d 1 to 21 and d 1 to 28, were significantly lower (*P* < 0.05) in broilers of the PC group. However, the addition of 300 mg/kg LTE significantly ameliorated the decline in growth performance induced by NE infection, as evidenced by a significant increase (*P* < 0.05) in BW on d 28 and ADG on d 1–28.

**Table 3:** Effects of LTE on growth performance of NE broilers

Item	NC	PC	LTE300	SEM	*P*-value
Body weight, g					
21 d	885.110^a^	808.823^b^	842.897^ab^	12.088	0.015
28 d	1,454.273^a^	1,365.764^b^	1,448.913^a^	14.202	0.002
1–21 d					
ADFI, g/d	59.740^a^	56.055^b^	59.003^ab^	0.735	0.082
ADG, g/d	40.623^a^	36.990^b^	38.613^ab^	0.575	0.015
FCR	1.475	1.518	1.528	0.013	0.243
Mortality, %	2.780	2.778	2.778	0.926	1.000
22–28 d					
ADFI, g/d	125.338	118.584	132.890	2.143	0.245
ADG, g/d	81.308	76.578	86.572	2.349	0.225
FCR	1.540	1.562	1.558	0.032	0.121
Mortality, %	1.111	5.427	4.453	0.962	0.160
1–28 d					
ADFI, g/d	76.825	73.240	78.582	1.062	0.103
ADG, g/d	50.795^a^	47.634^b^	50.603^a^	0.507	0.002
FCR	1.513	1.542	1.546	0.018	0.431
Mortality, %	3.705	7.410	6.483	1.454	0.585

*ADFI* Average daily feed intake, *ADG* Average daily gain, *FCR* Feed conversion ratio, *NC* Unchallenged and without additive, *PC* Challenged and without additives, *LTE300* Challenged and dietary supplementation with 300 mg/kg LTE, *SEM* Standard error of the mean (*n* = 6)

^a,b^Different lowercase letters in the same row indicate significant differences (*P* < 0.05)

**Fig. 1:**
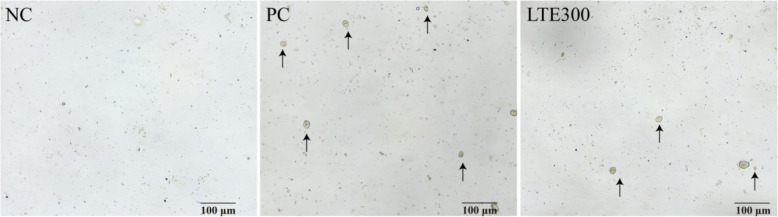
Representative image of coccidiosis oocysts in feces on d 18. Black arrows indicate coccidial oocysts. Scale bar: 100 μm. NC, unchallenged and without additive; PC, challenged and without additives; LTE300, challenged and dietary supplementation with 300 mg/kg LTE (*n* = 6)

**Fig. 2:**
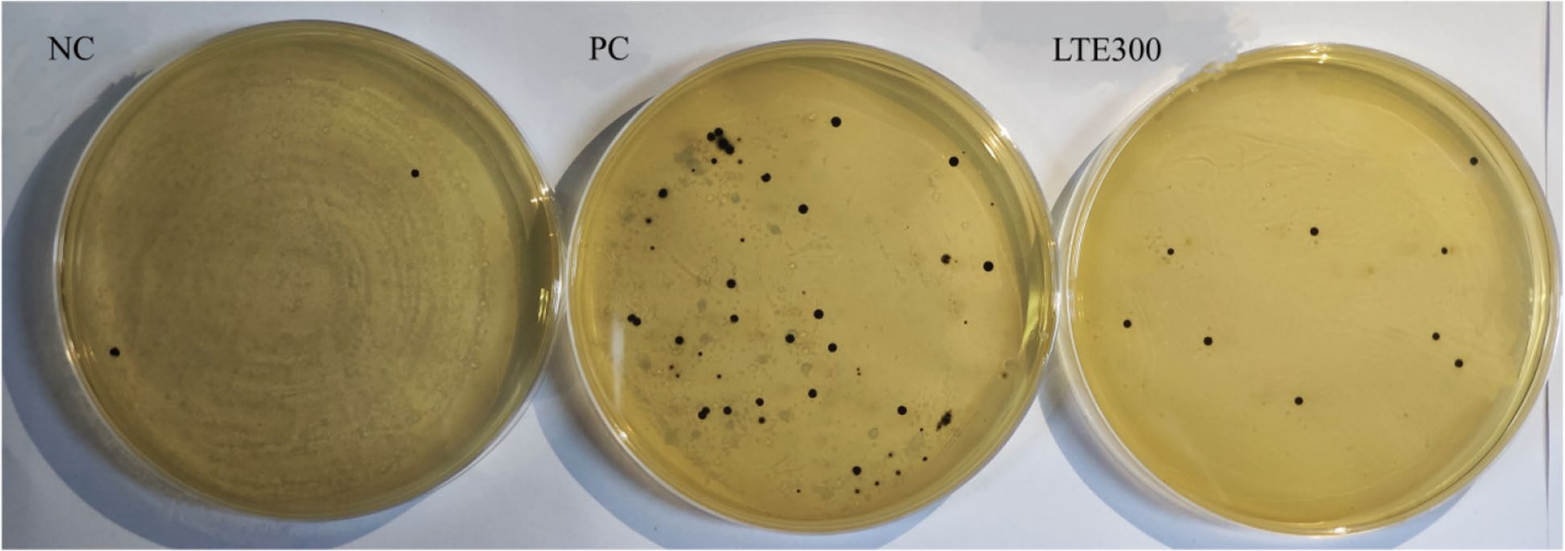
Effects of LTE on *Clostridium perfringens* in cecum of NE broilers on d 28 (Black dots indicate *Clostridium perfringens*). NC, unchallenged and without additive; PC, challenged and without additives; LTE300, challenged and dietary supplementation with 300 mg/kg LTE (*n* = 6)

The model of NE broiler was constructed by co-infection with coccidia and *Clostridium perfringens*. From d 5 to 7 (17–19 d) after coccidia infection, the number of coccidia oocysts in the PC group significantly increased (*P* < 0.05). The number of coccidia oocysts was significantly lower (*P* < 0.05) in the LTE300 group compared to the PC group (Table 4 and Fig. 1). The number of *Clostridium perfringens* in the intestine was further analyzed. It was found that the number of *Clostridium perfringens* was significantly increased (*P* < 0.05) in the PC group compared to the NC group. On the contrary, the addition of 300 mg/kg LTE to the diet significantly reduced the number of *Clostridium perfringens* (*P* < 0.05) (Table 4 and Fig. 2).

**Table 4:** Effects of LTE on fecal coccidia count and cecal *Clostridium perfringens* in NE broilers

Item	NC	PC	LTE300	SEM	*P*-value
Coccidia count					
17 d, × 10^4^ oocysts/g	-	8.60^a^	5.25^b^	0.669	0.002
18 d, × 10^4^ oocysts/g	-	4.30^a^	2.11^b^	0.441	0.001
19 d, × 10^4^ oocysts/g	-	1.85^a^	1.36^b^	0.126	0.037
*Clostridium perfringens* count					
21 d, lgCFU/g	5.81^b^	6.90^a^	6.13^b^	0.154	0.002
28 d, lgCFU/g	5.31^b^	6.92^a^	5.85^b^	0.215	< 0.001

*NC* Unchallenged and without additive, *PC* Challenged and without additives, *LTE300* Challenged and dietary supplementation with 300 mg/kg LTE, *SEM* Standard error of the mean; “-” indicates not detected (*n* = 6)

^a,b^Different lowercase letters in the same row indicate significant differences (*P* < 0.05)

Intestinal lesion score assessed the severity of NE. The results showed a significant increase in duodenum, jejunum, ileum and cecum in the PC group on d 21 compared to the NC group (*P* < 0.05). On d 21, the addition of 300 mg/kg LTE to the diet significantly reduced the increase in intestinal lesion score caused by NE (*P* < 0.05; Table 5).

**Table 5:** Effects of LTE on intestinal lesion score of NE broilers

Item	NC	PC	LTE300	SEM	*P*-value
21 d					
Duodenum	0.08^b^	0.67^a^	0.00^b^	0.093	0.001
Jejunum	0.00^b^	1.08^a^	0.16^b^	0.133	< 0.001
Ileum	0.00^b^	1.08^a^	0.08^b^	0.125	< 0.001
Cecum	0.08^b^	0.67^a^	0.00^b^	0.083	< 0.001
28 d					
Duodenum	0.08	0.75	0.13	0.149	0.102
Jejunum	0.38	0.88	0.88	0.145	0.264
Ileum	0.25	0.71	0.25	0.159	0.406
Cecum	0.08	0.25	0.00	0.053	0.150

*NC* Unchallenged and without additive, *PC* Challenged and without additives, *LTE300* Challenged and dietary supplementation with 300 mg/kg LTE, *SEM* Standard error of the mean (*n* = 6)

^a,b^Different lowercase letters in the same row indicate significant differences (*P* < 0.05)

### LTE inhibits intestinal inflammation and improves intestinal barrier function in NE broilers

**Fig. 3:**
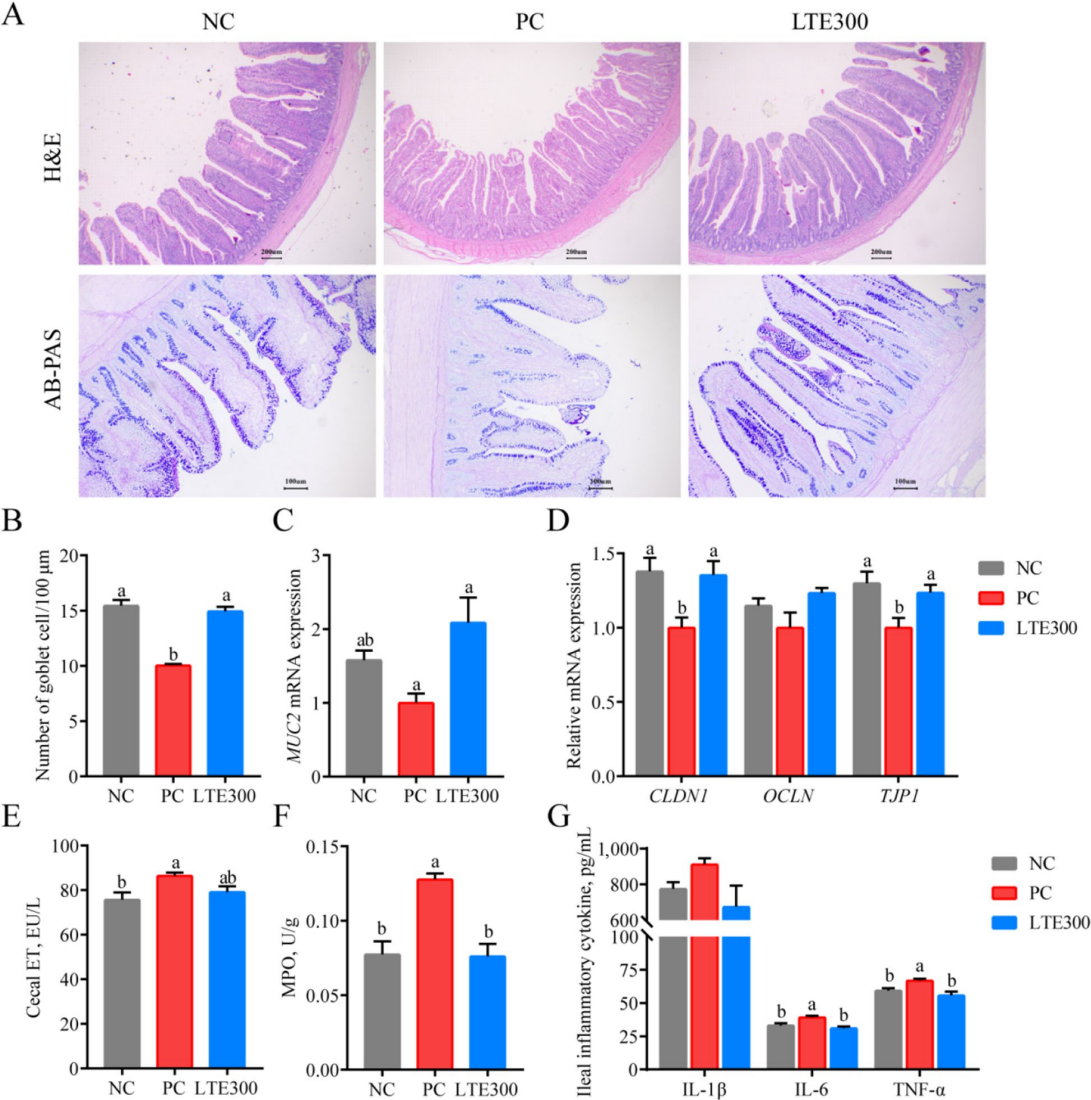
Effects of LTE on intestinal barrier function of NE broilers on d 28. **A** Representative images of ileum H&E and AB-PAS staining. **B** Number of ileal goblet cells. **C** *MUC2* mRNA expression level. **D** mRNA expression level of ileal tight junction-related genes. **E** Endotoxin content of cecal. **F** MPO content of ileum. **G** Inflammatory factor level of ileum. NC, unchallenged and without additive; PC, challenged and without additives; LTE300, challenged and dietary supplementation with 300 mg/kg LTE (*n* = 6). ^a,b^The different lowercase letters on the bar charts indicate significant differences (*P* < 0.05)

After determining the effect of LTE on intestinal macroscopic lesion scores, its effect on intestinal barrier structure and function was further investigated. Ileal CD was significantly increased (*P* < 0.05) and VH/CD was significantly decreased (*P* < 0.05) in the PC group. Compared with the PC group, we found that LTE300 significantly increased VH and significantly decreased CD, resulting in higher VH/CD (*P* < 0.05) (Table 6 and Fig. 3A). In addition, AB-PAS staining showed that LTE300 group significantly reversed the decrease in the number of ileal goblet cells and *MUC2* mRNA expression caused by NE (*P* < 0.05; Fig. 3A–C). suggesting that LTE300 promotes the improvement of mucus secretion and intestinal barrier function to some extent.

**Table 6:** Effects of LTE on ileum morphology of NE broilers on d 28

Item	NC	PC	LTE300	SEM	*P*-value
VH, μm	685.27^b^	597.03^b^	968.09^a^	46.871	< 0.001
VW, μm	187.38	188.85	185.75	3.856	0.955
CD, μm	115.30^b^	132.86^a^	115.07^b^	2.794	0.002
MH, μm	279.97	290.56	277.62	8.330	0.820
VH/CD	5.96^b^	4.50^c^	8.44^a^	0.478	< 0.001

*VH* Villus height, *VW* Villus width, *CD* Crypt depth, *MH* Muscular height, *NC* Unchallenged and without additive, *PC* Challenged and without additives, *LTE300* Challenged and dietary supplementation with 300 mg/kg LTE, *SEM* Standard error of the mean (*n* = 6)

^a–c^Different lowercase letters in the same row indicate significant differences (*P* < 0.05)

Tight junctions, which include proteins such as TJP1, CLDN1, and OCLN, are crucial for maintaining the function of the intestinal barrier. The mRNA expression levels of *CLDN1* as well as *TJP1* were significantly lower in the PC group (*P* < 0.05). LTE300 significantly increased the mRNA expression levels of *CLDN1* as well as *TJP1* compared to the PC group (*P* < 0.05; Fig. 3D). The cecal ET results also responded to the improvement of the intestinal barrier structure by LTE300 group, and the results showed that LTE300 reduced the elevated ET levels induced by NE (Fig. 3E). In addition, MPO, IL-6 and TNF-α levels were significantly increased in the PC group compared to the NC group (*P* < 0.05). However, this result was significantly reversed by LTE300 (*P* < 0.05; Fig. 3F and G).

### LTE alleviates oxidative stress and mitochondrial dysfunction in NE broilers

**Fig. 4:**
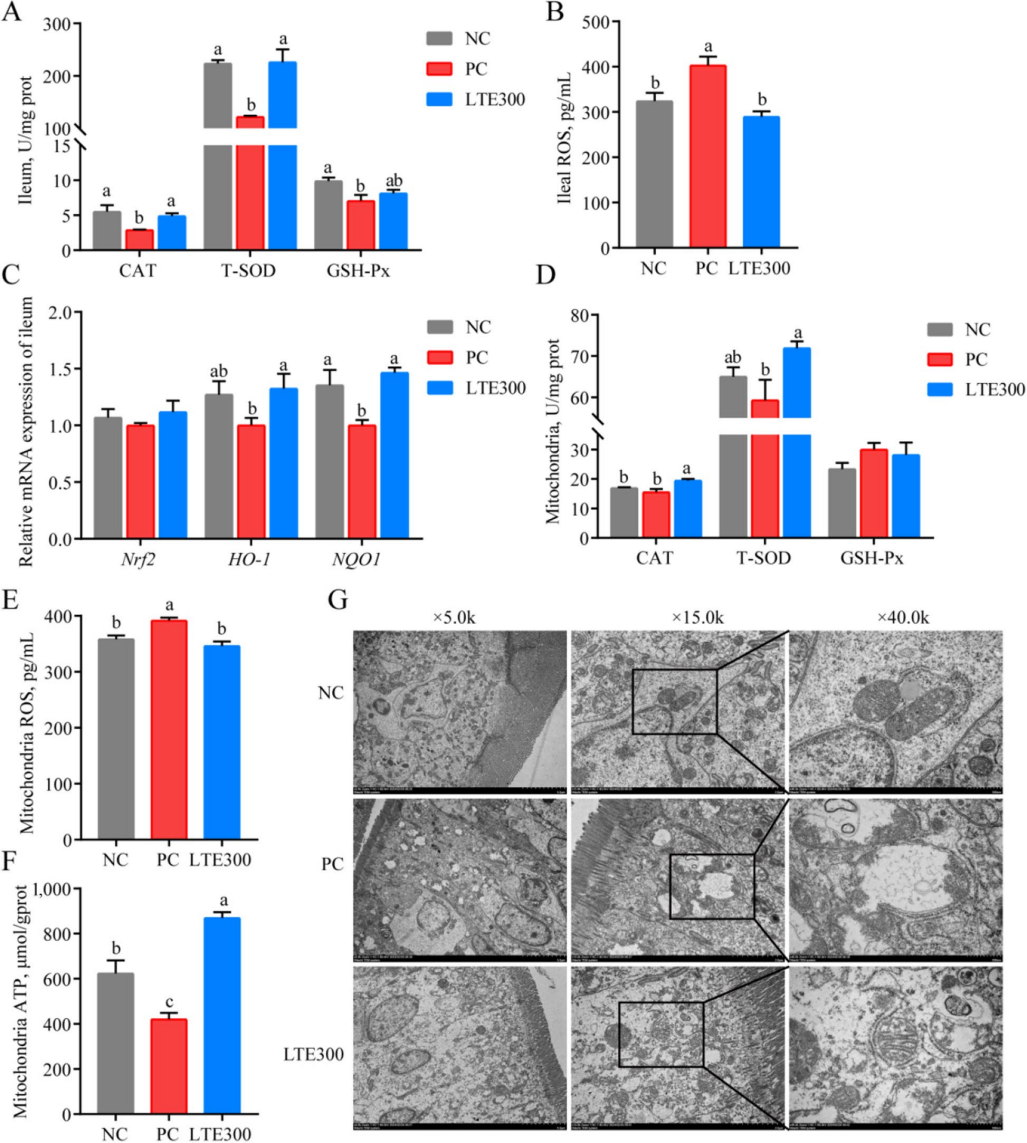
Effects of LTE on oxidative stress and mitochondrial function in the ileum of NE broilers on d 28. **A** CAT, T-SOD, and GSH-Px content of ileal tissue. **B** ROS content of ileal tissue. **C** *Nrf2*, *HO-1*, and *NQO-1* mRNA expression levels. **D** Mitochondrial CAT, T-SOD, and GSH-Px content. **E** ROS content of mitochondrial. **F** ATP content of mitochondrial. **G** Transmission electron micrographs of mitochondria (Scale bar: 5 μm, 2 μm, 500 nm; Magnification: × 5.0 k, × 15.0 k, × 40.0 k). NC, unchallenged and without additive; PC, challenged and without additives; LTE300, challenged and dietary supplementation with 300 mg/kg LTE (*n* = 6). ^a^^–^^c^The different lowercase letters on the bar charts indicate significant differences (*P* < 0.05)

Inflammation is closely related to oxidative stress. NE infection significantly decreased the levels of ileal antioxidant enzymes (CAT, T-SOD, and GSH-Px) in broilers (*P* < 0.05; Fig. 4A) and stimulated ROS production (*P* < 0.05; Fig. 4B). LTE300 significantly increased ileal CAT and T-SOD levels (*P* < 0.05) and significantly decreased ROS levels (*P* < 0.05) compared with the PC group. The mRNA expression level in the ileal Nrf2 pathway was further explored, and we found that LTE300 significantly reversed the reduction of *HO-1* and *NQO1* mRNA expression levels induced by NE infection (*P* < 0.05; Fig. 4C).

Moreover, oxidative stress causes mitochondrial damage, which subsequently contributes to the overproduction of ROS, ultimately leading to a vicious cycle. Therefore, we further investigated the mitochondrial structure and function. The results revealed that the mitochondrial CAT level in the LTE300 group was significantly higher than that in the NC and PC groups (*P* < 0.05), and the T-SOD level was also significantly higher in the LTE300 group compared to the PC group (*P* < 0.05; Fig. 4D). LTE300 group significantly reversed the increase in mitochondrial ROS levels (*P* < 0.05) and decrease in ATP levels (*P* < 0.05) induced by NE infection (Fig. 4E and F).

Results of transmission electron microscopy showed that the mitochondrial structure was intact and clear and neatly arranged in the NC group. After NE infection, the vast majority of mitochondria appeared swollen, cristae were reduced or disappeared, and membranes were disrupted; the morphology of mitochondria was almost completely enlarged and rounded, and the interior was vacuolated. However, after treatment with LTE, mitochondrial cristae and matrix density were more normal, and membranes were intact (Fig. 4G). These results suggest that LTE may alleviate mitochondrial dysfunction and oxidative stress in NE broilers by activating the Nrf2 signaling pathway and increasing the levels of antioxidant enzymes.

### LTE reducing endotoxin exposure and mitigating secondary liver injury in NE broilers

**Fig. 5:**
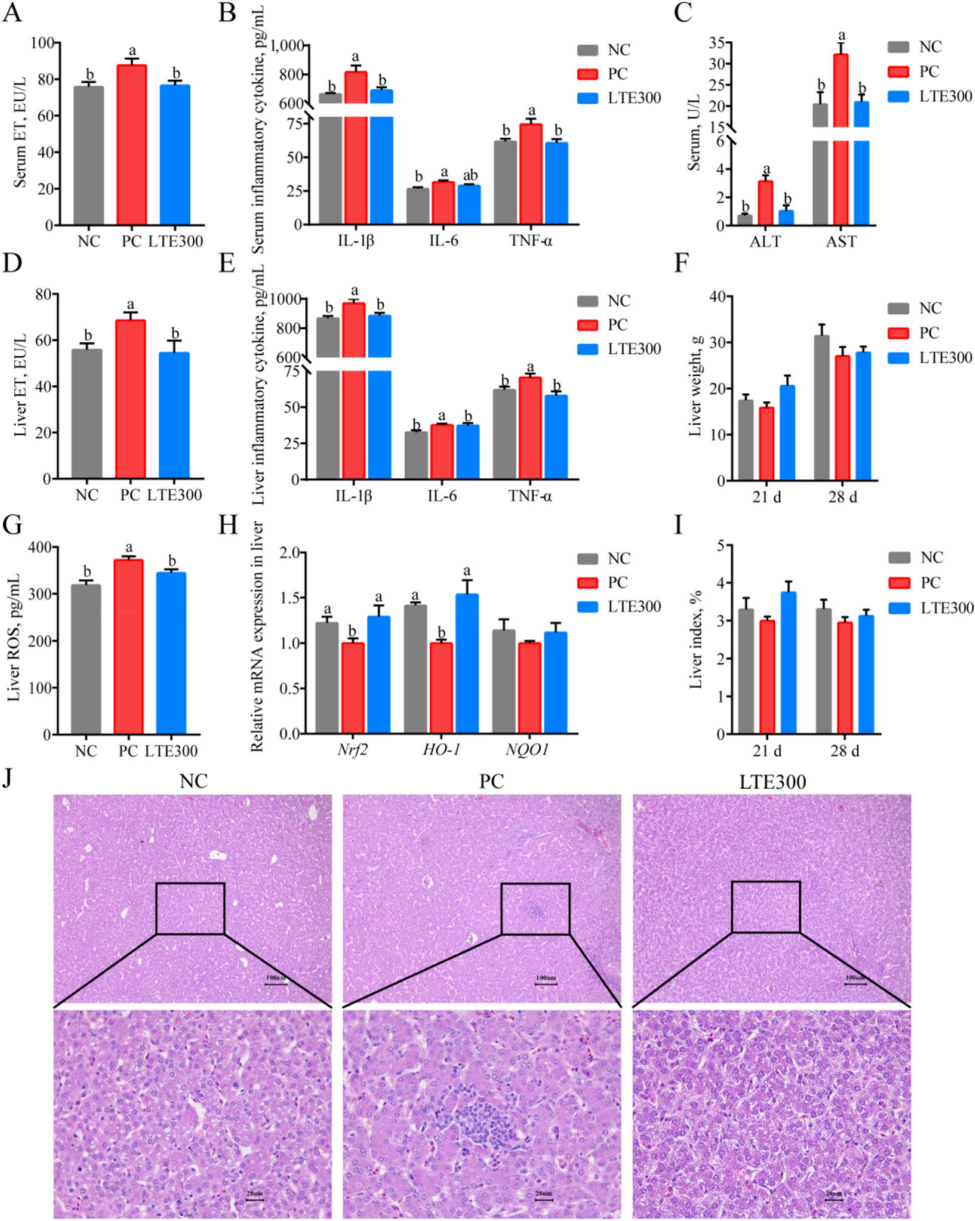
Effects of LTE on secondary liver injury in NE broilers on d 28. **A** Serum endotoxin levels. **B** Serum inflammatory cytokine levels. **C** Serum AST and ALT levels. **D** Liver endotoxin levels. **E** Liver inflammatory cytokine levels. **F** Liver weight. **G** Liver ROS levels. **H** *Nrf2*, *HO-1*, *NQO-1* mRNA expression levels. **I** Liver index. **J** Liver H&E staining. NC, unchallenged and without additive; PC, challenged and without additives; LTE300, challenged and dietary supplementation with 300 mg/kg LTE (*n* = 6). ^a,b^The different lowercase letters on the bar charts indicate significant differences (*P* < 0.05)

Disruption of the intestinal mucosal barrier leads to ET exposure. ET, IL-1β, IL-6 and TNF-α levels in serum were significantly elevated in the PC group compared to the NC group (*P* < 0.05). LTE300 treatment significantly reduced the elevated ET, IL-1β and TNF-α levels in NE broilers (*P* < 0.05) (Fig. 5A and B). Increased ET exposure due to compromised intestinal barrier predisposes the liver to secondary injury. The results showed a significant increase in serum levels of AST and ALT in the PC group, accompanied by an increase in liver ET and pro-inflammatory cytokines (IL-1β, IL-6 and TNF-α). However, AST, ALT, liver ET and liver pro-inflammatory cytokines were significantly reduced (*P* < 0.05) after LTE300 treatment (Fig. 5C, and E).

LTE300 group also reduced the increase in liver ROS levels induced by NE infection (*P* < 0.05; Fig. 5G). The Nrf2 signaling pathway is an important mechanism of cellular defence against oxidative stress and inflammatory responses, and NE infection significantly reduced *Nrf2* and *HO-1* mRNA expression levels, which were significantly increased after the addition of LTE300 (*P* < 0.05; Fig. 5H). H&E staining of the liver also showed that no morphological abnormalities were observed in the livers of broilers in the NC group, whereas foci of inflammatory cell infiltration were seen in the liver parenchyma of broilers after NE infection, which were mainly dominated by lymphocytes and epithelioid macrophages, with degeneration and necrosis of peripheral hepatocytes. There were no obvious abnormalities in broiler livers and the pathological changes were significantly ameliorated after LTE300 treatment (Fig. 5J). These results indicated that LTE alleviated SLI caused by NE. The ameliorative effect of LTE on SLI may be achieved by up-regulating the Nrf2 signaling pathway, inhibiting pro-inflammatory cytokines and reducing endotoxin levels.

### LTE improves the gut microbiota of NE broilers

**Fig. 6:**
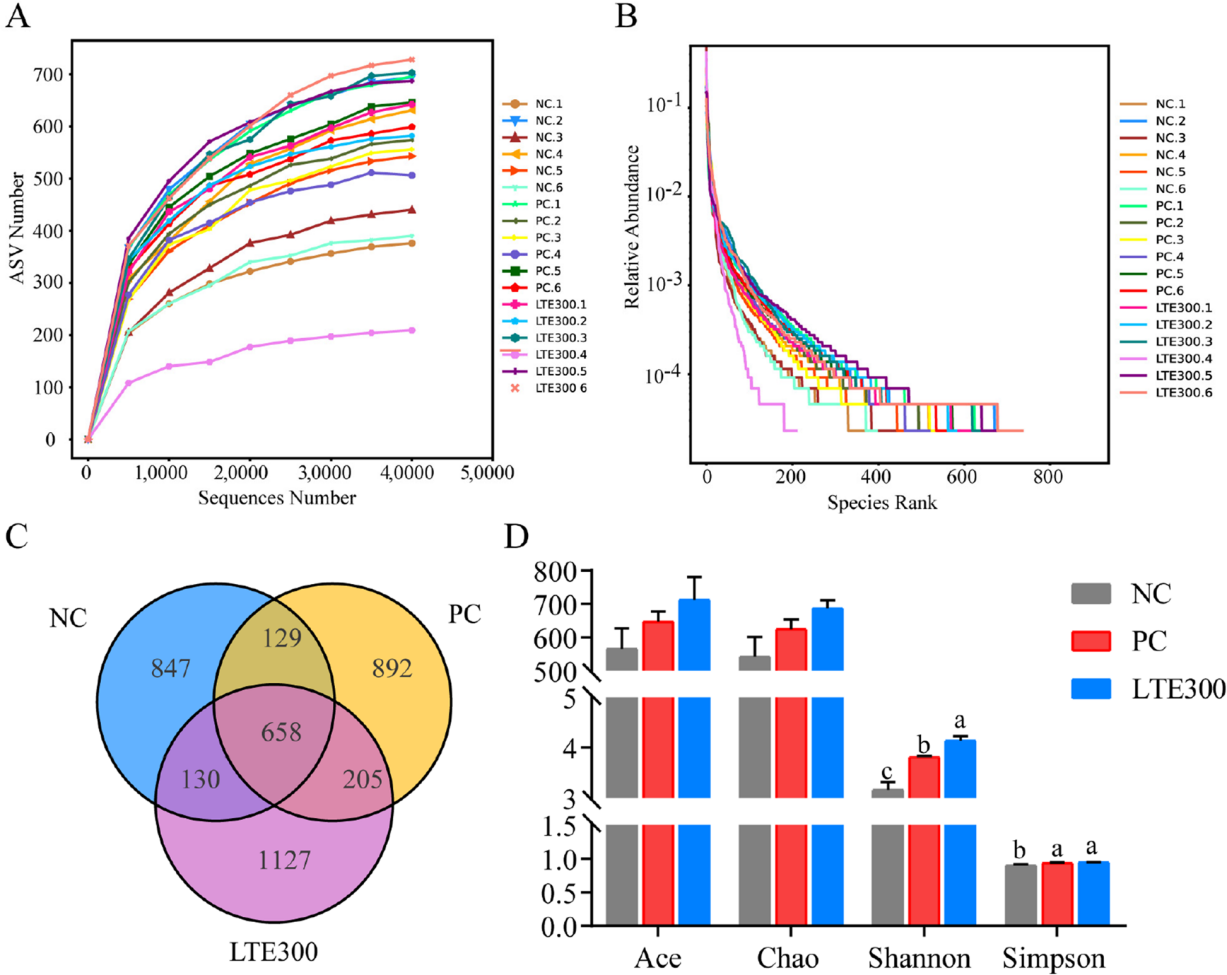
Effects of LTE on microbial diversity in the cecum of NE broilers on d 28. **A** Rarefaction curve. **B** Rank abundance curve. **C** Venn diagram. **D** The α-diversity parameters including ACE, Chao, Shannon, and Simpson indices. NC, unchallenged and without additive; PC, challenged and without additives; LTE300, challenged and dietary supplementation with 300 mg/kg LTE (*n* = 6). The different lowercase letters on the bar charts indicate significant differences (*P* < 0.05)

The effects of LTE on the cecal microbiota of NE broilers was further explored. Results showed that the rarefaction curve and rank abundance curve of all samples levelled off with the increase of sequencing volume, indicating that the sequencing depth had basically covered the vast majority of species in the samples to meet the needs of subsequent analyses (Fig. 6A and B). The Venn diagram results showed that the NC group had 847 specific OTUs, the PC group had 892 specific OTUs, and the LTE300 group contained 1,127 specific OTUs (Fig. 6C). Results for α diversity indicated that the Shannon index of the LTE300 group was significantly higher than that of the PC group (*P* < 0.05), and the Simpson index was significantly higher than that of NC group (*P* < 0.05) (Fig. 6D).

**Fig. 7:**
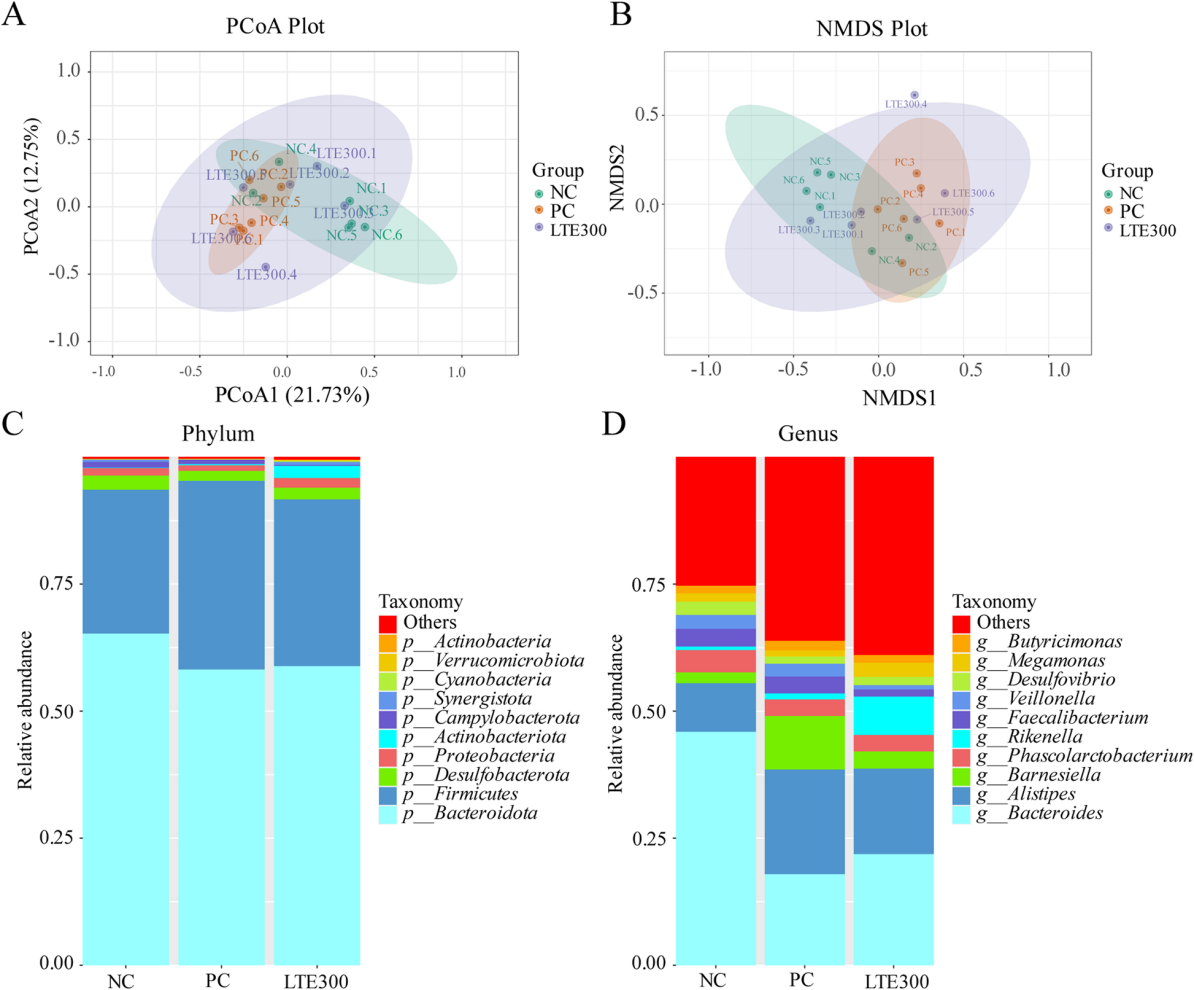
Effects of LTE on microbial community structure and composition in the cecum of NE broilers on d 28. **A** Principal co-ordinate analysis (PCoA) plot. **B** Non-metric multidimensional scaling (NMDS) plot. **C** Microbiota in the top 10 species of relative abundance at the phylum level. **D** Microbiota in the top 10 species of relative abundance at the genus leel. NC, unchallenged and without additive; PC, challenged and without additives; LTE300, challenged and dietary supplementation with 300 mg/kg LTE (*n* = 6)

Principal co-ordinates analysis (PCoA) and ANOSIM analyses showed that samples from the NC and PC groups were significantly separated. The LTE300 group exhibited greater sample dispersion and reduced the separation of gut microbiota caused by NE (Fig. 7A and Table 7). The non-metric multidimensional scaling NMDS results also indirectly support this (Fig. 7B). At the phylum level, Bacteroidota and Firmicutes were the main dominant phyla, accounting for more than 90% of the entire phylum level (Fig. 7C). At the genus level, *Bacteroides*, *Alistipes*, *Barnesiella*, *Phascolarctobacterium* and *Rikenella* dominated the cecum microbial community (Fig. 7D).

**Table 7:** ANOSIM for microbiota composition among the three treatments

Item	R-value	*P*-value
NC vs. PC	0.489	0.016
NC vs. LTE300	0.056	0.222
PC vs. LTE300	0.148	0.067

*R*-values range from −1 to 1. When *R*-values are > 0, the difference between groups is significant, and when *R*-values are < 0, the difference within groups is greater than the difference between groups. *NC* unchallenged and without additive, *PC* challenged and without additives, *LTE300* challenged and dietary supplementation with 300 mg/kg LTE (*n* = 6)

Further analyses were performed to determine statistical differences in microbiota among the groups. As shown in Table 8, the relative abundance of both Bacteroidota and Desulfobacterota was significantly lower (*P* < 0.05) in the PC group compared to the NC group. The relative abundance of Verrucomicrobiota in the LTE300 group was significantly increased (*P* < 0.05) compared to the PC group. As shown in Table 9, the PC group significantly decreased the relative abundance of *Bacteroides* and significantly increased the relative abundance of *Barnesiella* compared to the NC group (*P* < 0.05). However, LTE300 group significantly reduced the increase in relative abundance of *Barnesiella* caused by NE infection (*P* < 0.05).

**Table 8:** Effects of LTE on top 10 microbes on phylum level in the cecal of NE broilers on d 28

Item	NC	PC	LTE300	SEM	*P*-value
Bacteroidota	70.248^a^	62.228^b^	60.702^b^	1.276	0.001
Firmicutes	28.248	32.338	30.628	1.654	0.626
Desulfobacterota	3.282^a^	0.758^b^	2.305^ab^	0.408	0.029
Proteobacteria	1.450	1.097	0.760	0.134	0.104
Actinobacteriota	0.073	0.230	0.066	0.046	0.272
Campylobacterota	1.160	0.422	0.218	0.177	0.064
Synergistota	0.124	0.000	0.588	0.141	0.206
Cyanobacteria	0.030	0.087	0.143	0.019	0.050
Verrucomicrobiota	0.002^b^	0.005^b^	0.031^a^	0.005	0.009
Actinobacteria	0.002	0.110	0.000	0.023	0.066

*NC* Unchallenged and without additive, *PC* Challenged and without additives, *LTE300* Challenged and dietary supplementation with 300 mg/kg LTE, *SEM* Standard error of the mean (*n* = 6)

^a,b^Different lowercase letters in the same row indicate significant differences (*P* < 0.05)

**Table 9:** Effects of LTE on top 10 microbes on genus level in the cecal of NE broilers on d 28

Item	NC	PC	LTE300	SEM	*P*-value
Bacteroides	54.606^a^	17.924^b^	21.861^b^	5.083	0.001
Alistipes	9.563	20.587	16.827	2.798	0.277
Barnesiella	2.117^b^	6.394^a^	2.285^b^	0.663	0.025
Phascolarctobacterium	4.392	3.254	3.269	0.620	0.717
Rikenella	0.697	1.182	0.712	0.343	0.821
Faecalibacterium	1.440	3.316	1.365	0.427	0.093
Veillonella	2.728	0.649	0.883	0.501	0.183
Desulfovibrio	2.609	1.402	1.618	0.495	0.598
Megamonas	1.645	1.209	1.045	0.367	0.810
Butyricimonas	1.472	1.875	1.535	0.271	0.826

*NC* Unchallenged and without additive, *PC* Challenged and without additives, *LTE300* Challenged and dietary supplementation with 300 mg/kg LTE, *SEM* Standard error of the mean (*n* = 6)

^a,b^Different lowercase letters in the same row indicate significant differences (*P* < 0.05)

### Spearman correlation heatmap

**Fig. 8:**
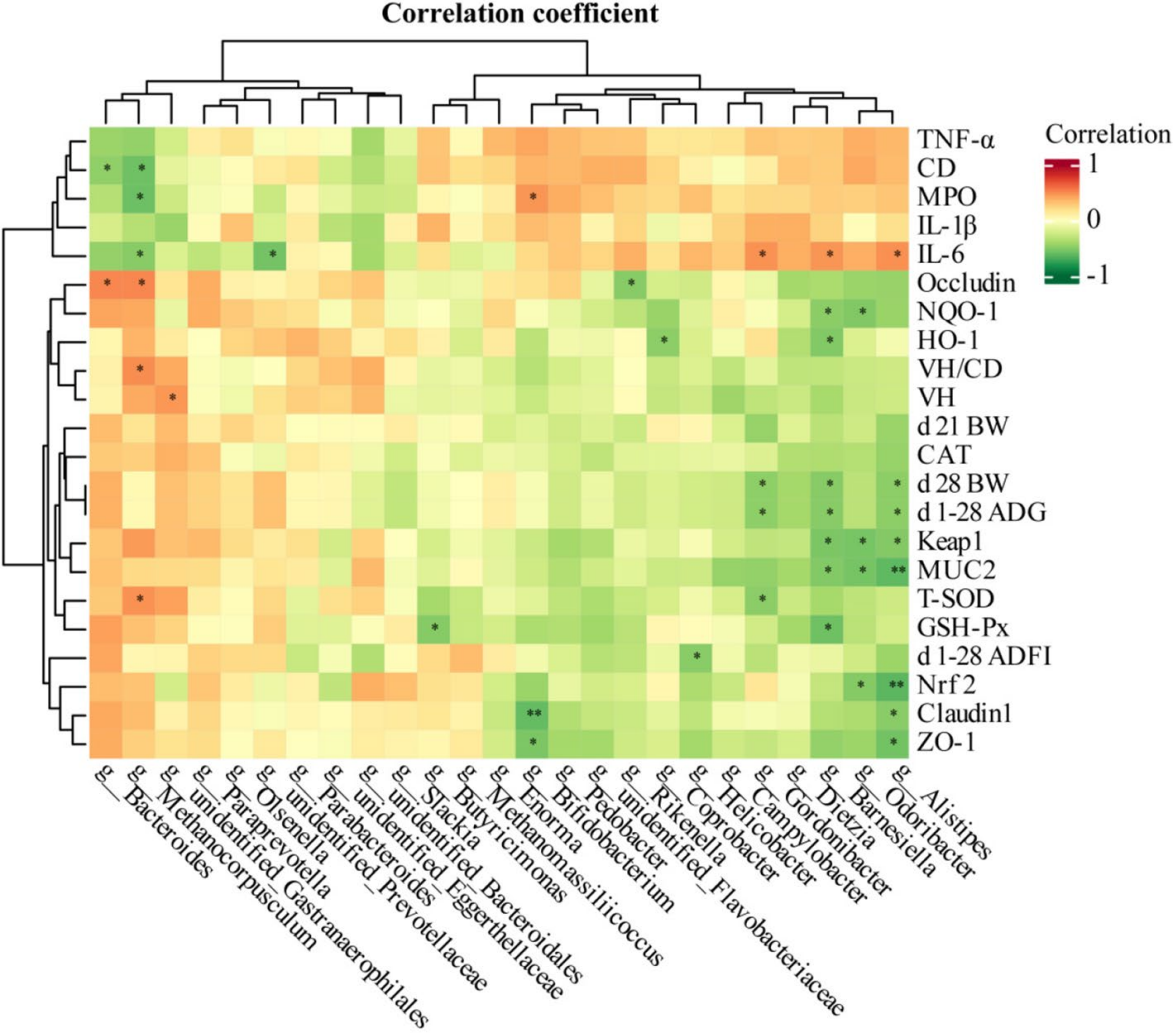
Heatmap of the correlation analysis between intestinal microbiota (at the genus level) parameters related to growth performance and intestinal barrier function on d 28. ^*^P < 0.05, ^**^P < 0.01

Spearman’s correlation analysis was used to explore the correlation between microbiota at the intestinal genus level and growth performance and intestinal barrier function (Fig. 8). The results showed that *Bacteroides* were significantly positively correlated with *OCLN* mRNA expression level and significantly negatively correlated with CD of intestinal (*P* < 0.05). *Methanocorpusculum* showed a significant positive correlation with ileal T-SOD, VH/CD, and *OCLN* mRNA expression levels, and a significant negative correlation with IL-6, MPO, and CD (*P* < 0.05). *Butyricimonas* showed a significant negative correlation with ileal GSH-Px levels (*P* < 0.05). *Rikenella* showed a significant negative correlation with *OCLN* mRNA expression level (*P* < 0.05). In addition, both *Barnesiella* and *Alistipes* showed significant positive correlation with IL-6 (*P* < 0.05). Both *Barnesiella* and *Alistipes* were significantly negatively correlated with broiler d 1–28 ADG, d 28 BW, ileal GSH-Px levels, and *MUC2* mRNA expression levels (*P* < 0.05).

## Discussion

Necrotic enteritis is a severe threat to poultry intestinal health and is a typical inflammatory bowel disease, causing significant economic losses to the global poultry industry. Numerous studies have shown that CA and CUR can effectively regulate intestinal barrier function and improve gut health [20, 23, 30, 31]. This study is the first to reveal the protective effects of LTE on NE and SLI. LTE can improve growth performance, reduce intestinal inflammation, and mitigate pathological changes in the liver. Additionally, LTE regulates intestinal barrier dysfunction by modulating the microbiota and mitochondrial dysfunction. The improvement in barrier function reduces gut-derived lipopolysaccharide levels, thereby further alleviating oxidative damage and inflammation in the liver.

Co-infection with coccidia and *Clostridium perfringens* is the most common method for inducing NE [32]. In this study, broilers co-infected with coccidia and *Clostridium perfringens* exhibited clear clinical symptoms of NE, including reduced body weight, impaired intestinal morphology, and increased levels of inflammatory cytokines and ROS, consistent with previous reports [14, 33]. It has been reported that the number of *Clostridium perfringens* in the intestines of healthy broilers usually ranges from 10^2^ to 10^4^ CFU/g, while in diseased broilers, the numbers can reach 10^7^ to 10^9^ CFU/g [34]. In this study, the number of *Clostridium perfringens* in NE broilers reached 10^7^ CFU/g. These results indicate the successful establishment of the necrotic enteritis model. Compared to the PC group, the use of LTE reduced intestinal damage scores, decreased the number of coccidia oocysts, and declined *Clostridium perfringens*, suggesting the potential of LTE in ameliorating NE. The current findings are supported by many previous studies that have demonstrated the inhibitory effects of CA and CUR on coccidia oocysts and their antimicrobial properties [35–37]. These studies further indicate the effectiveness of CA and CUR in combating coccidia invasion and inhibiting the growth of pathogenic bacteria.

Although previous studies have explored the effects of CA and CUR, research on their impact in NE infection models remains limited. In our prior study, LTE linearly increased the ADG of healthy broilers throughout the trial period (21–60 d) [18]. Dietary supplementation with 500 mg/kg CA has been reported to improve the growth performance of NE broilers [5]. CUR has also been shown to enhance the growth performance of broilers under various challenging conditions, such as diets contaminated with aflatoxin B1 [38], high stocking density [39], heat stress [40], and coccidia infection [41]. Consistent with these findings, the present study demonstrated that dietary supplementation with LTE improved body weight and ADG from 1 to 28 d in NE broilers. The improvement in growth performance may be attributed to the improved intestinal barrier function, alleviation of inflammation and oxidative stress, and modulation of gut microbiota by LTE.

The intestinal mucosal barrier, comprising the physical barrier, chemical barrier, immune barrier, and microbial barrier, is a crucial defense mechanism that protects the body from external harmful substances [42]. Increasing evidence suggests that dysfunction of the intestinal mucosal barrier is a key factor in the pathogenesis of NE [4]. The disruption of the mucosal barrier in NE leads to pathogen invasion and toxin accumulation, triggering intestinal inflammation and damage, which significantly impact overall health [6]. The physical barrier is formed by intestinal epithelial cells and tight junctions [43]. Intestinal morphology, an important indicator of digestive and absorptive function, shows abnormal changes in NE broilers, which are significantly improved by LTE supplementation. Furthermore, LTE supplementation markedly increased goblet cell numbers and *MUC2* mRNA expression levels, restoring the mucus layer in NE broilers and enhancing intestinal barrier function. Tight junctions, composed of key proteins such as TJP1, CLDN1, and OCLN, play a critical role in maintaining epithelial barrier integrity and intestinal permeability [43]. Xu et al. [14] observed significantly reduced mRNA expression levels of *TJP1* and *OCLN* in NE broilers. Similar results were found in this study, where NE infection led to down-regulation of *CLDN1* and *TJP1* mRNA expression. In contrast, dietary LTE significantly up-regulated the mRNA expression levels of *TJP1* and *CLDN1* in NE broilers, suggesting that LTE plays a positive role in restoring and enhancing tight junction structures. The improvement in tight junction structures reduces the risk of invasion by harmful substances, lowering intestinal permeability [44]. This was further confirmed by the decreased levels of ET in the intestines and serum.

Inflammatory responses are a hallmark of NE, and a highly activated inflammatory response can further disrupt intestinal homeostasis. MPO levels reflect the activation of neutrophils and the degree of inflammation [9]. Studies have found that NE broilers have significantly increased serum levels of MPO, lipopolysaccharide, and IL-6, along with up-regulated pro-inflammatory cytokine genes in the intestines [7]. Consistent with these findings, we observed high levels of MPO, IL-6, and TNF-α in the ileum of NE broilers, which were significantly reduced by LTE supplementation. Numerous studies have also reported the anti-inflammatory effects of CA and CUR [35, 36, 38, 39, 45], further supporting our results. Inflammation is closely associated with oxidative stress, which may play a critical role in the pathogenesis of NE. A considerable body of evidence indicates that the antioxidant capacity of NE broilers is significantly reduced [6, 7, 46]. Mitochondria are the primary sites of ROS production and also serve as the energy metabolism centers of cells [15]. Increased oxidative stress can damage mitochondria, and subsequent mitochondrial dysfunction can generate more ROS, creating a vicious cycle [5]. Therefore, regulating oxidative stress and mitochondrial function is crucial for alleviating NE. Previous studies have shown that LTE can regulate the antioxidant capacity of broilers by enhancing the activity of antioxidant enzymes in the serum and ileum [18]. Zhou et al. [30] reported that the protective effect of CA against endotoxin-induced intestinal injury in rats was associated with improved mitochondrial function and increased antioxidant enzyme activity. In vitro experiments also demonstrated that CA ameliorated H₂O₂-induced mitochondrial damage and ROS levels in the intestine, confirming its protective effect on mitochondria [47]. Additionally, CUR has been reported to improve oxidative stress-induced jejunal injury and mitochondrial dysfunction in piglets [31]. Consistent with these studies, our findings revealed that LTE significantly increased the levels of CAT, GSH-Px, and T-SOD in the ileum of NE broilers, significantly increased mitochondrial CAT and T-SOD levels, and reduced ROS levels in both the ileum and mitochondria, thereby alleviating intestinal oxidative stress. In addition, the CAT activity in the LTE group was significantly higher than that in the NC group, possibly because LTE may promote the synthesis of CAT or enhance its activity through some mechanism. The combined results from mitochondrial electron microscopy and ATP levels indicated that LTE improved the mitochondrial structure and function in NE broilers. In summary, these results indicate that LTE ameliorates mitochondrial dysfunction and oxidative damage in the intestines.

The disruption of the intestinal barrier can lead to the leakage of toxic and harmful substances from the intestinal lumen, with gut-derived ET being key factors in inducing liver injury [48]. Many reports have revealed that intestinal-derived lipopolysaccharide is closely associated with extraintestinal organ inflammation [9, 48–50], which was confirmed by our finding of significantly elevated levels of lipopolysaccharide in the cecum, serum, and liver in the PC group. Liver injury is a common extraintestinal manifestation of NE, and liver-derived inflammatory factors due to liver damage can further exacerbate necrotic enteritis [8, 51]. In our experiment, the PC group showed liver inflammatory cell infiltration and degenerative necrosis, with obvious pathological changes. Combined with elevated AST and ALT levels, and increased levels of inflammatory (IL-1β, IL-6, and TNF-α) and ROS, it was evident that the PC group had severe liver injury and inflammation. However, supplementation with LTE reduced AST, ALT, and lipopolysaccharide levels, alleviating the degree of liver injury and inflammation, consistent with previous results [19, 45, 52]. LTE also alleviated liver pathological changes, reduced serum and liver inflammatory cytokines (IL-1β, IL-6, and TNF-α), and decreased ROS levels. Stimulation by lipopolysaccharide promotes ROS production, which further aggravates tissue damage and inflammatory responses [50]. The Nrf2 signaling pathway is a crucial mechanism for cellular defense against oxidative stress and inflammation [8]. Under normal conditions, *Nrf2* is bound by *Keap1*, but under oxidative stress, *Nrf2* dissociates from *Keap1* and rapidly translocates into the nucleus to initiate the expression of a series of downstream antioxidant factors (*HO-1*, *NQO1*, etc.) [8]. It has been reported that activation of the Nrf2 signaling pathway can reduce oxidative stress levels [36, 49]. In this study, the PC group showed significantly down-regulated mRNA expression levels of *Nrf2* and *HO-1*. In contrast, LTE restored the mRNA expression levels of *Nrf2* and *HO-1* in the liver. This is consistent with the findings of Ji et al. [45], who demonstrated that CA protected meat rabbits from heat stress-induced liver injury by enhancing the Nrf2/HO-1 pathway. Liu et al. [36] indicated that CA improved growth performance and intestinal health in broilers under oxidative stress by activating the Nrf2 pathway. Other studies have also shown that CUR can activate the Nrf2/HO-1 pathway to ameliorate liver oxidative stress and inflammation levels [53]. In summary, the ameliorative effect of LTE on liver may be achieved by up-regulating the Nrf2 signaling pathway, inhibiting pro-inflammatory cytokines and reducing endotoxin levels.

Gut microbiota plays a crucial role in the host's nutrient metabolism, immune system development, and maintenance of gut barrier function, thereby significantly contributing to host health and intestinal homeostasis [10, 15]. Dysbiosis of the gut microbiota is closely related to the pathogenesis of NE. The results of this study indicate that NE infection significantly increased the Shannon and Simpson indices, which is consistent with previous research [54, 55]. The dynamic changes in the gut microbiota are influenced by pathogen infection, host immune response, and environmental factors [56]. We speculate that the infection of NE may increase the colonization and proliferation of microorganisms in the intestinal microbiota of broilers. However, for NE infection, some studies have found no significant changes in the Shannon and Simpson indices [7, 57], while some studies observed a decrease [13, 58]. This discrepancy may be related to differences in sample sources (such as ileum and cecum) and the timing of sample collection. Previous research has shown that NE infection increases the relative abundance of *Barnesiella* and *Alistipes* while decreasing the relative abundance of *Desulfobacterota*, *Synergistota*, *Bacteroides*, and *Anaerotruncus* in the cecum of chickens [13]. In this study, NE significantly increased the relative abundance of *Barnesiella* in the cecum of chickens, with correlation results showing a significant positive correlation between *Barnesiella* and IL-6. The increase in *Barnesiella* may be due to the favorable conditions for anaerobic bacteria created by *Clostridium perfringens* infection [13], indirectly reflecting dysbiosis in the gut microbiota. Additionally, NE significantly decreased the relative abundance of *Bacteroidota*, *Desulfobacterota*, and *Bacteroides* in the cecum. *Bacteroidota* and *Bacteroides* can digest complex carbohydrates (cellulose and resistant starch), providing nutrients for the host or other microbes [56]. Propionate and butyrate are significant metabolic products of these bacteria and can regulate inflammatory responses and intestinal homeostasis [13, 56]. Correlation results showed that *Bacteroides* was significantly positively correlated with *OCLN* mRNA expression. Therefore, the reduction in *Bacteroidota* and *Bacteroides* might trigger imbalances in gut homeostasis and immune system regulation. *Desulfobacterota*, a class of sulfate-reducing bacteria, can metabolize to produce hydrogen sulfide [59], and its reduction may be related to changes in the redox state within the gut. Dysbiosis of the gut microbiota can lead to gut barrier dysfunction and persistent inflammation. Dietary LTE has been proven to improve gut health by modulating the gut microbiota and immune function [18]. In this study, LTE increased the OTUs and Shannon index in NE chickens, and in combination with ANOSIM analysis, LTE intake restored the separation of the gut microbiota caused by NE infection. Additionally, LTE increased the relative abundance of *Verrucomicrobiota* in the gut. Most representatives of *Verrucomicrobiota* in the gut belong to *Akkermansia muciniphila*, a beneficial bacterium that can improve the host's metabolic functions and immune responses [11]. These results suggest the beneficial role of LTE in enhancing microbial diversity and restoring gut microbiota balance.

## Conclusion

In conclusion, the findings suggest that LTE may alleviate NE and SLI by enhancing intestinal barrier function and regulating intestinal microbiota imbalance, thereby improving gut-liver axis health as well as growth performance. This suggests that regulation of gut-liver axis health should not be overlooked when treating NE.

## Data Availability

The data analyzed during the current study are available from the corresponding author upon reasonable request. The 16S rRNA sequencing data have been deposited into the NCBI Sequence Read Archive database (accession number: PRJNA1268728).

## Abbreviations

AB-PAS: Alcian blue-Periodic acid Schiff
ADFI: Average daily feed intake
ADG: Average daily gain
ALT: Alanine aminotransferase
AST: Aspartate aminotransferase
ATP: Adenosine triphosphate
BW: Body weight
CA: Chlorogenic acid
CAT: Catalase
CD: Crypt depth
CLDN1: Claudin 1
CUR: Curcumin
ELISA: Enzyme-linked immunosorbent assay
ET: Endotoxin
FCR: Feed conversion ratio
GSH-Px: Glutathione peroxidase
H&E: Hematoxylin and eosin
HO-1: Heme oxygenase-1
IL-1β: Interleukin-1β
IL-6: Interleukin-6
LTE: Lonicerae flos and turmeric extracts
LTE300: Challenged and dietary supplementation with 300 mg/kg LTE
MH: Muscular height
MPO: Myeloperoxidase
MUC2: Mucin 2
NC: Unchallenged and without additive
NE: Necrotic enteritis
NQO1: NAD(P)H quinone oxidoreductase-1
Nrf2: Nuclear erythroid-derived 2-related factor 2
OCLN: Occludin
OTUs: Operational taxonomic units
PC: Challenged and without additive
PCoA: Principal co-ordinates analysis
qRT-PCR: Quantitative real-time PCR
ROS: Reactive oxygen species
SCFA: Short-chain fatty acid
SEM: Standard error of the mean
SLI: Secondary liver injury
TJP1: Tight junction protein 1
TNF-α: Tumor necrosis factor-α
T-SOD: Total superoxide dismutase
VH/CD: The ratio of villus height to crypt depth
VH: Villus height
VW: Villus width
